# TeaGVD: A comprehensive database of genomic variations for uncovering the genetic architecture of metabolic traits in tea plants

**DOI:** 10.3389/fpls.2022.1056891

**Published:** 2022-11-28

**Authors:** Jie-Dan Chen, Wei-Zhong He, Si Chen, Qi-Yu Chen, Jian-Qiang Ma, Ji-Qiang Jin, Chun-Lei Ma, Doo-Gyung Moon, Sezai Ercisli, Ming-Zhe Yao, Liang Chen

**Affiliations:** ^1^ National Center for Tea Improvement, Tea Research Institute of the Chinese Academy of Agricultural Science, Hangzhou, China; ^2^ Tea Research Institute, Lishui Academy of Agricultural and Forestry Sciences, Lishui, China; ^3^ Research Institute of Climate Change and Agriculture, National Institute of Horticultural and Herbal Science, Jeju, South Korea; ^4^ Department of Horticulture, Faculty of Agriculture, Ataturk University, Erzurum, Turkey

**Keywords:** tea plant, genomic variation, database, genotype-to-phenotype associations, metabolite

## Introduction

Tea plant (*Camellia sinensis* (L.) O. Kuntze) is one of the most important nonalcoholic beverage crops. As a result of distinctive metabolites beneficial to health, tea plant is also widely used to uncover the molecular mechanisms underlying the synthesis of specific metabolites, such as catechins and caffeine (Jin et al., 2017; Jiang et al., 2019; Zhu et al., 2021). The rapid development of high-throughput sequencing technologies has led to an exponential increase in the volume of biological sequence data of tea plants over the past decade, providing valuable insights into the diversity and evolution of tea germplasms and the mechanism of important metabolites and agronomic traits in tea plant. Tea genome is large (~3 Gb) and complex, harboring a large number of repetitive sequences and high heterozygosity due to its self-incompatibility. Recently, the completion and availability of genome assemblies of tea plant have accelerated the investigations of evolutionary dynamics of whole-genome duplication, tandem duplication, and long terminal repeat retrotransposons that resulted in the diversification of tea germplasms (Wang et al., 2020; Xia et al., 2020; Chen et al., 2020; Zhang et al., 2020a; Zhang et al., 2020c; Wang et al., 2021; Zhang et al., 2021). Meanwhile, large-scale resequencing and RNA-seq projects of tea germplasms have been performed and enabled novel insights into the diversity, evolution and domestication in tea germplasms (Wang et al., 2020; Xia et al., 2020; Yu et al., 2020; Zhang et al., 2020c; Zhang et al., 2021). Genome-wide linkage study and genome-wide association study (GWAS) have revealed numerous sites and genes controlling relevant agronomical traits of tea plant, such as leaf traits (An et al., 2021; Lu et al., 2021) and metabolites (Zhang et al., 2020b), which provide an important foundation for further decoding the molecular mechanism of traits in tea plant.

However, the lack of a standardized data processing and visualizing platform hinders the availability of such data. The construction of a user-friendly web-based platform for big data deposition, integration, accession and visualization has become a crucial issue for maximizing these valuable sequence data. Recently, several specialized web-based databases have been developed for the storage and utilization of biological sequence data in tea plant, such as TPIA (Xia et al., 2019), TeaPGDB (Lei et al., 2021), TeaCoN (Zhang et al., 2020b), and TeaAS (Mi et al., 2021). However, these databases did not comprehensively integrate a large-scale genomic variation of various tea genetic resources and genotype-to-phenotype associations (G2Ps) for understanding the complex traits in tea plants, hindering the availability of big omics data. Here, we collected and identified more than 70 million genomic variations and 17,974 high-quality G2Ps for 464 tea metabolites. A comprehensive and user-friendly database of genomic variations for tea plants, TeaGVD (http://www.teaplant.top/teagvd), has been developed for storage, retrieval, visualization and utilization of these data, which will facilitate understanding of the genetic architecture of metabolic and agronomic traits, molecular assistant breeding, and molecular design breeding in tea plants.

## Materials and methods

### Data sources

Currently, the raw reads of whole-genome sequencing (WGS), GBS data and RNA-seq data from eight datasets of tea germplasms comprising 1,229 accessions were collected (**Table S1**). All the species and varieties in *Camellia* L. Sect. *Thea* (L.) Dyer were covered, including *C. sinensis* (L.) O. Kuntze var. *sinensis*, var. *assamica* (Masters) Kitamura, var. *pubilimba* Chang, *C. taliensis* (W. W. Smith) Melchior, *C. tachangensis* F. C. Zhang, *C. crassicolumna* Chang, and *C. gymnogyna* Chang (Chen et al., 2000). Four datasets of WGS germplasms representing genetic diversity and improvement of tea plants were downloaded from NCBI with BioProject accession numbers PRJNA646044, PRJNA597714, PRJNA665594, and PRJNA716079 (Wang et al., 2020; Xia et al., 2020; Lu et al., 2021; Zhang et al., 2021). GBS data were downloaded from the Genome Sequence Archive in National Genomics Data Center, China National Center for Bioinformation/Beijing Institute of Genomics, Chinese Academy of Sciences with CRA001438 (Niu et al., 2020). Other datasets were RNA-seq data and downloaded from PRJNA595795 and PRJNA562973 with 217 and 136 tea accessions, respectively (Yu et al., 2020; Zhang et al., 2020c). In addition, GA and eight catechin compounds in three leaf samples of 176 tea accessions (Zhang et al., 2020c) and 437 annotated metabolites detected by UPLC-QTOF MS of 136 tea accessions (Yu et al., 2020) were integrated into the database. Because a high-quality chromosome-level genome assembly is basis for identification of genomic variations and genome-wide association analysis, the reference genome (*C. sinensis* var. *sinensis* ‘Shuchazao’), functional annotation and gene expression were downloaded from the Tea Plant Information Archive (http://tpdb.shengxin.ren/) (Xia et al., 2019). Two previously published draft genomes of *C. sinensis* var. *sinensis* ‘Shuchazao’ and *C. sinensis* var. *assamica* ‘Yunkang 10’ have widely applied in genetic and functional studies in tea plants (Xia et al., 2017; Wei et al., 2018). For users’ convenience, a total of 31,780 orthologous gene sets were identified for the three tea genome assemblies by using BLASTP (Altschul et al., 1997) based on the bidirectional best hit (BBH) method (**Table S2**).

### Data processing

To identify the genomic variation of tea germplasms accurately, the raw reads were trimmed by Sickle (https://github.com/najoshi/sickle) with default parameters to remove low-quality sequences. In WGS germplasms, the trimmed reads were aligned to the tea pant reference genome using Burrows Wheeler Aligner (BWA) (Li and Durbin, 2009) and PCR duplicates were filtered by Sambamba (Tarasov et al., 2015) with parameters “–overflow-list-size 1000000 –hash-table-size 1000000”. After filtering low-quality alignments, SNP and InDel were identified by SAMtools (Li et al., 2009) and FreeBayes (Garrison and Marth, 2012). In GBS germplasms, the trimmed reads were aligned to the tea pant reference genome using BWA (Li et al., 2009) and SNP and InDel were identified by HaplotypeCaller of GATK with parameters “–minimum-mapping-quality 30 -ERC GVCF –dont-use-soft-clipped-bases” (McKenna et al., 2010). In RNA-seq germplasms, the trimmed RNA-seq reads were mapped to the reference genome using HISAT2 with default parameters (Kim et al., 2019). PCR duplicates were removed by Picard (https://broadinstitute.github.io/picard). SNP and InDel calling was performed by HaplotypeCaller of GATK (McKenna et al., 2010). These SNPs and InDels were further filtered by VCFtools with parameters “–max-missing 0.5 –minQ 30 –maf 0.05” (Danecek et al., 2011). The identified genomic variations were annotated by SnpEff (Cingolani et al., 2012), ANNOVAR (Wang et al., 2010) and VEP (McLaren et al., 2016) based on the gene annotation file of the tea plant genome with default parameters.

To explore the genetic diversity of tea germplasms, the SNP density, nucleotide diversity (*θπ*), and Tajima’s D statistics of 461 WGS germplasms were calculated by VCFtools (Danecek et al., 2011). In addition, GWAS was performed with EMMAX (Kang et al., 2010) and GAPIT (Wang and Zhang, 2021) with GLM, MLM, CMLM and FarmCPU model to find genetic variations or genes associated with a particular metabolic trait. The threshold of significant candidate loci (lead SNPs) was determined by GEC software (Li et al., 2012). The LD Score regression intercept and heritability were estimated by LDSC software (https://github.com/bulik/ldsc).

### Implementation

The interactive web interface of TeaGVD was built based on Flask, a lightweight Python Web framework (https://palletsprojects.com/p/flask/), and it integrated all pre-processed data. The frontend pages were developed and visualized by HTML5, CSS5, jQuery, Bootstrap (https://getbootstrap.com/), ECharts (https://echarts.apache.org/), and Bokeh (https://bokeh.org/). The BLAST tool was implemented using SequenceServer (Priyam et al., 2019). In the PCR primer design tool, Primer3 (Koressaar and Remm, 2007) was used to pick PCR primers based on the reference genome with customization.

## Database contents

**Figure 1:**
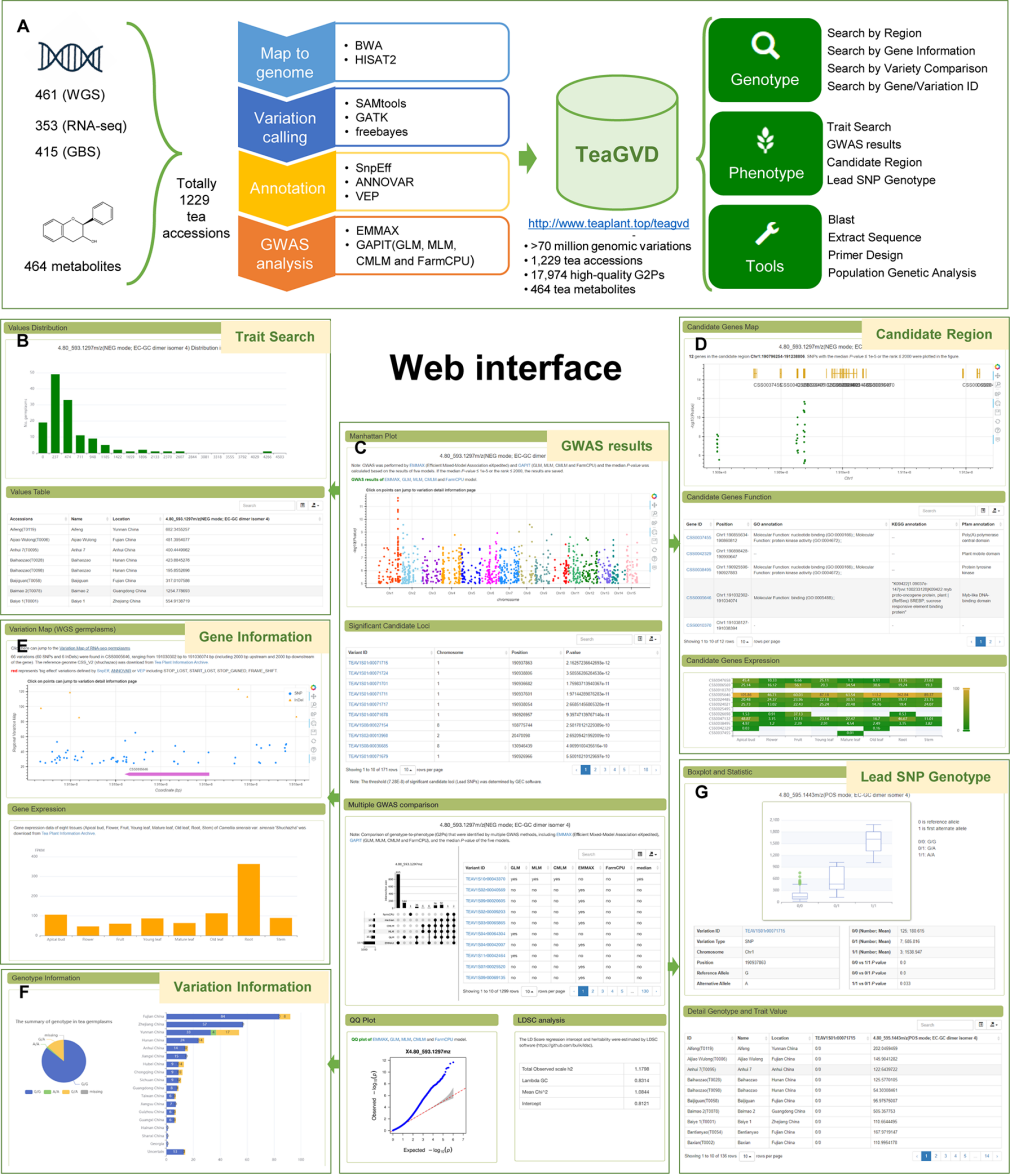
The schematic, screenshots of representative resources in the TeaGVD. **(A)** The schematic of TeaGVD. **(B)** The distribution of trait value and detailed value in the available tea accessions. **(C)** The Manhattan plot, QQ plot, and significant candidate loci of GWAS for EC-GC dimer isomer 4. **(D)** The distribution, function, and expression of candidate genes in the candidate region (Chr1:190796254-191238806) that identified by GWAS. **(E)** The detail gene information and variation map of the candidate gene CSS0005646. **(F)** The genotype distribution of the significant candidate loci TEAV1S01r00071715. **(G)** Boxplot and statistic analysis of different genotypes in TEAV1S01r00071715 for EC-GC dimer isomer 4.

To take advantages of omics data in tea plant, the sequencing data of 1,229 accessions of tea germplasms were collected and analyzed using a standardized pipeline. In total, more than 70 million genomic variations (SNPs and InDels) were identified from the sequencing data (**Table 1**). The missing rate and level of heterozygosity were 20.27% and 16.73%, respectively. Among these, 6,193,642, 30,938, and 944,449 genomic variations were present in or around gene regions (e.g., exon, intron, upstream, and downstream), accounting for 8.74%, 17.66% and 77.15% of these in WGS, GBS, and RNA-seq data, respectively. In addition, 17,974 high-quality G2Ps for 464 tea metabolites have been identified by GWAS. To facilitate the exploration of these data, we developed a comprehensive and user-friendly database of genomic variations in tea plants (TeaGVD) that was built and organized into three functional modules for various data types and applications, including Genotype, Phenotype, and Tools modules (**Figure 1A**). These modules provide user-friendly web interfaces to retrieve and visualize genomic variations and their related information. In the Genotype module, users can retrieve available SNP/InDel information by multiple search strategies with filter parameters. Moreover, TeaGVD can figure out the polymorphic SNPs/InDels between two or more germplasms rapidly by comparison of varieties, which is convenient to develop molecular markers. In the Phenotype module, TeaGVD shows the detailed trait values, value distribution, and GWAS results for each available metabolite. Users also can further explore candidate genes and functional markers associated with the metabolite of interest by the Candidate Region and Lead SNP Genotype submodules, respectively. To better utilize these data, the BLAST, Extract Sequence, Primer Design, and Population Genetic Analysis (SNP density, nucleotide diversity, and Tajima’s D statistics) tools were established in the Tools module.

**Table 1:** Statistics of genomic variations and genotype-to-phenotype associations for metabolites in tea plants.

	WGS Germplasms		GBS Germplasms		RNA-seq Germplasms		Metabolites
	SNP	InDel	SNP	InDel	SNP	InDel	G2Ps
Chr1	5,222,352	145,700	15,105	638	90,750	5,014	1,760
Chr2	4,969,532	138,453	12,520	585	90,360	5,013	1,558
Chr3	4,482,574	123,313	10,230	436	84,345	3,860	1,427
Chr4	4,646,325	126,954	12,721	557	76,367	4,124	1,464
Chr5	4,673,005	121,241	10,392	450	64,876	3,090	1,634
Chr6	4,086,500	117,347	10,738	513	75,703	4,050	1,754
Chr7	4,473,987	118,746	10,999	473	75,863	3,838	896
Chr8	4,022,025	100,780	10,857	447	49,403	2,284	757
Chr9	3,869,937	105,272	10,179	462	70,129	3,616	966
Chr10	4,006,236	106,247	9,073	394	58,059	2,810	688
Chr11	2,921,987	82,127	8,403	394	59,777	3,451	1,209
Chr12	3,814,285	101,332	9,220	389	48,865	2,520	674
Chr13	3,139,356	87,970	8,158	378	57,964	3,112	762
Chr14	2,985,432	85,252	8,134	381	54,914	2,925	1,310
Chr15	2,811,586	77,317	6,736	280	45,377	2,411	1,115
UN	8,859,214	246,973	14,293	648	162,059	7,287	–
Total	68,984,333	1,885,024	167,758	7,425	1,164,811	59,405	17,974

## Use cases

These data and tools will facilitate understanding of the genetic architecture of metabolic traits and molecular breeding in tea plants. We take EC-GC dimer isomer 4 under NEG mode as an example. Histogram plot of value distribution and table of detail value for each tea germplasm are shown by selecting the corresponding trait in Trait Search (**Figure 1B**). GWAS results present the Multiple GWAS comparison, GWAS Manhattan plot, QQ plot, LDSC analysis, and significant candidate loci (lead SNP) associated with EC-GC dimer isomer 4, which can be dynamically visualized by clicking on given SNP/InDel links to various detailed information pages of variation (**Figure 1C**). On the basis of the GWAS results, we specified genomic coordinate (Chr1:190796254-191238806) in Candidate Region and identified 12 genes in the genomic region. The gene distribution, functional annotation, and expression of these genes are displayed in the web interface (**Figure 1D**). The given gene links direct users to gene detailed information interface, which includes a visualized variation map around the gene, basic gene information, gene annotation (GO, KEGG, and Pfam), and gene expression of eight tissues (**Figure 1E**). Among these, CSS0005646 (also known as *CsMYB111*) has been reported to be associated with anthocyanin, catechin, and flavanol biosynthesis (Li et al., 2022). In addition, TEAV1S01r00071715 significantly associated with EC-GC dimer isomer 4 (*P-value* < 2.16e-12) was identified by GWAS. Comparisons of different genotypes in TEAV1S01r00071715 showed that the content of EC-GC dimer isomer 4 of genotypes AA and AG was significantly higher than that of genotype GG (*P-value* < 0.01, two-sided Wilcoxon test; **Figure 1G**) by lead SNP genotyping. We also found that genotype AA was only present in Yunnan, China, which was the center of origin for tea plants (**Figure 1F**).

## Data availability statement

The datasets presented in this study can be found in online repositories. The names of the repository/repositories and accession number(s) can be found in the article/**Supplementary material** and http://www.teaplant.top/teagvd.

## Author contributions

LC, M-ZY, J-DC, and W-ZH conceived and designed the study. J-DC, SC, Q-YC, J-QM, J-QJ and C-LM. performed the data analysis and web design. J-DC, W-ZH, LC, M-ZY, D-GM and SE prepared the manuscript. All authors contributed to the article and approved the submitted version.

## Funding

This research was supported by the National Key Research and Development Program of China (2021YFD1200203), the Zhejiang Provincial Natural Science Foundation of China (LQ20C160010), the Zhejiang Science and Technology Major Program on Agricultural New Variety Breeding-Tea Plant (2021C02067) and the Fundamental Research Fund for Tea Research Institute of the Chinese Academy of Agricultural Sciences (1610212022009).

## Conflict of interest

The authors declare that the research was conducted in the absence of any commercial or financial relationships that could be construed as a potential conflict of interest.

## Publisher’s note

All claims expressed in this article are solely those of the authors and do not necessarily represent those of their affiliated organizations, or those of the publisher, the editors and the reviewers. Any product that may be evaluated in this article, or claim that may be made by its manufacturer, is not guaranteed or endorsed by the publisher.
